# Cross-Activation of the Motor Cortex during Unilateral Contractions of the Quadriceps

**DOI:** 10.3389/fnhum.2017.00397

**Published:** 2017-08-03

**Authors:** Ashlee M. Hendy, Lilian Chye, Wei-Peng Teo

**Affiliations:** ^1^Institute for Physical Activity and Nutrition (IPAN), School of Exercise and Nutrition Science, Deakin University Burwood, VIC, Australia; ^2^Frailty Research Programme, Geriatric Education and Research Institute Yishun Central, Singapore

**Keywords:** transcranial magnetic stimulation, quadriceps, bilateral-transfer, cross-education, motor evoked potential

## Abstract

Transcranial magnetic stimulation (TMS) studies have demonstrated that unilateral muscle contractions in the upper limb produce motor cortical activity in both the contralateral and ipsilateral motor cortices. The increase in excitability of the corticomotor pathway activating the resting limb has been termed “cross-activation”, and is of importance due to its involvement in cross-education and rehabilitation. To date, very few studies have investigated cross-activation in the lower limb. Sixteen healthy participants (mean age 29 ± 9 years) took part in this study. To determine the effect of varying contraction intensities in the lower limb, we investigated corticomotor excitability and intracortical inhibition of the right rectus femoris (RF) while the left leg performed isometric extension at 0%, 25%, 50%, 75% and 100% of maximum force output. Contraction intensities of 50% maximal force output and greater produced significant cross-activation of the corticomotor pathway. A reduction in silent period duration was observed during 75% and 100% contractions, while the release of short-interval intracortical inhibition (SICI) was only observed during maximal (100%) contractions. We conclude that increasing isometric contraction intensities produce a monotonic increase in cross-activation, which was greatest during 100% force output. Unilateral training programs designed to induce cross-education of strength in the lower limb should therefore be prescribed at the maximal intensity tolerable.

## Introduction

It is well established that unilateral muscle contractions of the upper limb are associated with bilateral activity of the motor cortex (M1; Carson, 2005). Whilst unilateral movements are primarily driven by activity in the contralateral M1 (cM1), activation of the ipsilateral M1 (iM1) has been demonstrated with transcranial magnetic stimulation (TMS; Muellbacher et al., 2000; Perez and Cohen, 2008; Howatson et al., 2011) and functional magnetic resonance imaging (Kobayashi et al., 2003; van Duinen et al., 2008). This increase in iM1 excitability measured by TMS has been termed “cross-activation”, and is of particular interest due to its potential role in the phenomenon of cross-education (Ruddy and Carson, 2013). Cross-education occurs following repetitive unilateral activities such as motor skill or strength training, with lasting performance improvements in the untrained limb believed to be mediated by persistent adaptations in the iM1 (Goodwill et al., 2012; Hendy et al., 2014; Leung et al., 2015).

Studies in the upper limb have demonstrated that the magnitude of cross-activation is greatest during high or maximal intensity contractions (Muellbacher et al., 2000; Perez and Cohen, 2008), while lower contraction intensities have yielded mixed results. One early study demonstrated that isometric contractions of the first dorsal interosseous (FDI) as low as 10% of the maximum voluntary contraction (MVC) have been shown to produce facilitation of the iM1 motor evoked potential (MEP) recorded from the contralateral FDI (Stedman et al., 1998). In contrast, another study found that intensities of greater than 50% MVC are required to elicit MEP facilitation in abductor pollicis brevis (Muellbacher et al., 2000). High intensity unilateral contractions in the upper limb nearly abolish short-interval intracortical inhibition (SICI) in the iM1 (Muellbacher et al., 2000; Perez and Cohen, 2008), indicating cross-activation is primarily of cortical origin. However, lower level contraction intensities have not been investigated during neither upper nor lower limb contractions. The effects of unilateral contraction on the cortical silent period, which represents inhibition at both spinal and suprasinal levels, are unknown. At present, there is no data to indicate the intensity of unilateral contractions required to facilitate excitability or reduce inhibition in the iM1 and corticomotor pathway of the lower limb. This knowledge is critical when considering the prescription of unilateral exercise to induce cross-education (Dragert and Zehr, 2013; Kim et al., 2015).

The majority of research into cross-activation has focused on unilateral movements of the upper limbs, with few studies examining the potential for this effect in the lower limb (Chiou et al., 2013a,b). Both these studies showed that forceful unilateral contractions of the rectus femoris (RF) and tibialis anterior (TA) did indeed produce cross-activation of the iM1, evidenced by increased corticomotor excitability, and a reduction in SICI and interhemispheric inhibition (IHI; Chiou et al., 2013a,b). The authors noted several unique properties of bilateral connectivity in the lower limbs, most notably the lack of homologous muscle-dominance, which is widely reported in upper limb musculature (Chiou et al., 2013a). This included an increase in corticomotor excitability and reduction of SICI recorded from the RF during contractions of the anterior deltoid and flexor carpi radialis (FCR; Chiou et al., 2013a). The differences in bilateral limb co-ordination during functional tasks between lower and upper limbs may influence the nature of cross-activation. For example, effortful upper limb tasks such as lifting typically require co-activation of homologous muscle groups, whereas movements in the lower limbs often involve muscle pairs working in a reciprocal manner (such as gait). Further investigation of the properties of cross-activation specific to the lower limb is warranted, particularly when considering the potential for cross-activation and cross-education to be utilized in unilateral lower limb rehabilitation settings such as anterior cruciate ligament (ACL) reconstruction and knee replacement (Hendy et al., 2012; Chiou et al., 2013a).

It is also possible that the anatomical location of the muscle investigated may influence the magnitude of cross-activation. In animals, ipsilateral projections to the distal forelimb are less pronounced (Soteropoulos et al., 2011). One study in humans has reported ipsilateral MEPs present in the biceps, finger and wrist extensors, but weak or absent in the triceps, opponens pollicis, finger and wrist flexors (Ziemann et al., 1999). Collectively, these results indicate that the influence of unilateral contractions on the inactive corticomotor pathway is specific to the muscle group tested, providing a rationale for further investigation of the phenomenon in the quadriceps.

The primary aim of this study was to determine the effects of unilateral isometric contractions of the non-dominant (left) quadriceps at 25%, 50%, 75% and 100% of MVC, on the cross-activation of the iM1 and corticomotor pathway, as determined from MEPs recorded from the right RF during both resting and low level contractions. It was hypothesized that high intensity isometric contractions would result in an increase in the excitability of the corticomotor pathway of the resting quadriceps along with a subsequent decrease in SICI of the iM1 and reduction in the silent period.

## Materials and Methods

### Participants

Sixteen right-footed participants (10 males and 6 females; age (mean ± standard deviation (SD) 29 ± 9 years) with no recent lower limb injuries volunteered for the study. A medical questionnaire was used to screen the participants for neurological disorders and contraindications in relation to the application of TMS. This study was carried out in accordance with the recommendations of the Deakin University Human Research Ethics Committee with written informed consent from all subjects. All subjects gave written informed consent in accordance with the Declaration of Helsinki. The protocol was approved by the Deakin University Human Research Ethics Committee.

### Experimental Design

**Figure 1:**
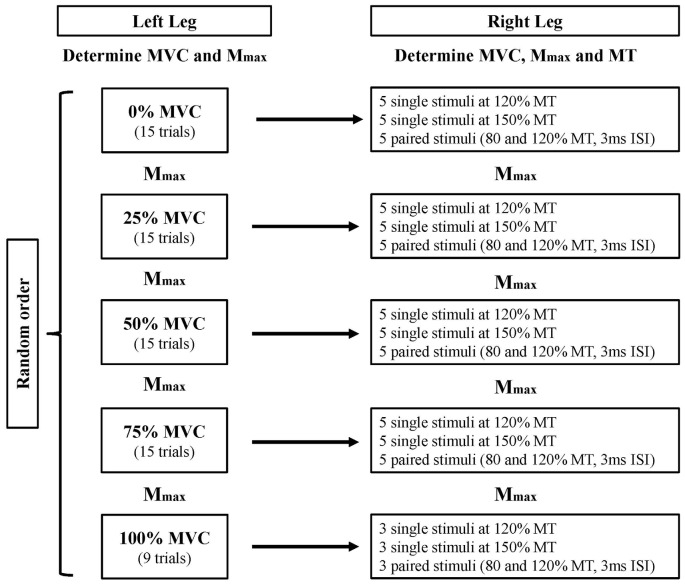
Experiment design. The left quadriceps contracts at five different contraction intensities (0, 25, 50, 75 and 100% MVC) while corticomotor responses of the right rectus femoris (RF) were measured. The measurement order of left quadriceps contraction intensity and transcranial magnetic stimulation (TMS) stimuli intensity were randomized. The entire protocol was first completed for *Experiment 1* (right leg resting), then repeated for *Experiment 2* (right leg extended). MVC, maximal voluntary contraction; *M*_max_, maximal compound wave; MT, motor threshold; ISI, interstimulus interval.

The participants attended a single laboratory session, where they performed isometric contractions with the left quadriceps while corticomotor responses were recorded from the right RF. The participant was seated in the isokinetic dynamometer (Biodex System Pro 4, Shirley, NY, USA) for the duration of the experiment (all measures). The participants were required to contract their left quadriceps at 25%, 50%, 75% and 100% of their predetermined MVC, in a randomized order (see “Voluntary Contractions during Testing” Section for details). While the participants were performing left quadriceps contractions, the corticomotor excitability of the right RF were measured when the right leg was relaxed (*Experiment 1*). In order to measure the duration of the silent period, the muscle of interest must be engaged in voluntary contraction. To achieve this, the same measures were conducted while the right leg was in full knee extension (*Experiment 2*). In accordance with previous literature, any trials in *Experiment 1* exhibiting background electromyography (EMG) activity above 0.025 mV for the period 50 ms prior to stimulus delivery were discarded, and the trial was repeated after reminding the participant to relax the right leg (Muellbacher et al., 2000). This was monitored online by an automated function in the EMG software (ADInstruments, Bella Vista, NSW, Australia) that provided the maximal peak-to-peak EMG value for the right leg during each pre-stimulus period. In addition, examination of pre-stimulus root mean squared (rms) EMG for the period 50 ms prior to stimulus delivery was used to monitor any difference in background muscle activity in the right leg during the left leg contraction tasks, for both experiments. Maximum compound muscle action potentials (*M*_max_) of the right and left RF were measured after each contraction intensity to assess peripheral fatigue. Single and paired-pulse TMS were delivered to the left M1 in a randomized order to produce MEPs in the right RF, to assess the cross-activation effects produced by the increasing contraction intensities of the left quadriceps. Figure 1 shows the experimental design.

### Maximum Voluntary Contraction (MVC)

Maximal voluntary contractions of the left quadriceps was measured using an isokinetic dynamometer (Biodex System Pro 4, Shirley, NY, USA) at the beginning of the experiment. The dynamometer arm was positioned to achieve 45° of knee flexion and was secured to the participants’ ankle with a velcro strap. Participants executed 3–5 submaximal isometric contractions at 10%–50% of their perceived MVC as a warm-up. The participants were then asked to extend with maximum effort, sustaining the isometric contractions for 3 s. All participants performed three trials of MVC separated by 3 min rest, with the highest force output recorded in newton. Verbal encouragement and visual feedback were given for each trial.

### Voluntary Contractions during Testing

During the experiment, the participants performed isometric contraction of the quadriceps at five different intensities with their left leg (0%, 25%, 50%, 75% and 100% MVC). The order of the contraction intensity was randomized for each participant. Online visual feedback of force production was provided to allow participant and researcher to monitor and achieve accurate force production for each trial. The required force output was manually set with a red line using the dynamometer software (Biodex System Pro 4, Shirley, NY, USA), and was visible on a computer screen located 1 m away from the participant at eye level. Participants were instructed to extend the left leg and maintain a constant force to match the red line for at least 2 s. The experimenter applied a TMS stimulus on the left M1 when the required force output was observed on the screen (determined visually by the experimenter). During *Experiment 1*, the participants were reminded at regular intervals to relax the right leg to minimize co-contraction. During *Experiment 2*, the participants were asked to extend the right leg straight in front of them (full extension) and maintain this position, with short rests provided after each stimulus.

### Maximum Compound Muscle Action Potential (*M*_max_)

Supramaximal electrical stimulation was applied to the left and right femoral nerves to obtain muscle responses (M-waves) from RF using a DS7A constant current electrical stimulator (Digitimer, Hertfordshire, UK), while the participant remained at rest. The back support of the seat was reclined 45° from the upright position to aid in the placement of electrode at the femoral triangle level beneath the inguinal ligament. The optimal intensity of stimulation for recruitment of all RF motor units was determined when the increasing current intensity did not result in any further increase of the EMG response. To ensure a maximal response, the current strength was further increased by another 10%, and five stimuli were delivered with an inter-stimulus period of 6–9 s. The largest peak-to-peak response from each RF (left and right) was reported as *M*_max_. The test was administered at the beginning of the session, and following the completion of all trials at each contraction intensity, with the resulting *M*_max_ used to normalize all MEP responses to ensure that any changes in peripheral muscle excitability did not influence corticomotor responses (Carroll et al., 2002).

### Transcranial Magnetic Stimulation

Single and paired-pulse TMS was used to assess the corticomotor excitability and SICI of the motor cortical representation of the right RF muscle. A 110 mm double-cone coil was used to elicit an MEP from the right RF muscle via the left M1 using a BiStim unit attached to two Magstim 200^2^ stimulators (Magstim Co, Dyfed, UK). The optimal site to elicit the largest and most consistent MEP response in the right RF was determined by exploring the leg representation of left M1 1–3 cm lateral and anterior of the vertex, and was marked with a felt tipped pen to ensure consistent coil placement throughout the experiment. The interval between delivery of each pulse varied based on the participants ability to perform left leg contractions, but was always greater than 10 s. The pre-stimulus rmsEMG activity of the right RF was recorded 50 ms prior to each TMS stimulus to monitor background muscle activity. For *Experiment 1*, resting motor threshold (RMT) was defined as the minimum stimulus intensity that produced an MEP response of >50 μV in 5 out of 10 consecutive trials while the right leg remained at rest. To quantify corticomotor excitability, five single-pulse stimuli were delivered at stimulator output intensities 120% RMT and 150% RMT. To quantify SICI, five paired-pulse stimuli were delivered with a conditioning stimulus of 80% RMT, a test stimulus of 120% RMT and an interstimulus interval (ISI) of 3 ms. For *Experiment 2*, the active motor threshold (AMT) was defined as the minimum stimulus intensity that produced an MEP response of >200 μV in 5 out of 10 consecutive trials while the right leg was fully extended (0° of knee flexion). The same protocols (120% and 150% AMT corticomotor excitability, and SICI) were then performed using equivalent stimulator output intensities and ISI. The MEPs resulting from single-pulse stimulation at 120% and 150% AMT during *Experiment 2* were used to quantify silent period. Responses from single-pulse stimuli delivered at 120% and 150% AMT were used to assess silent period duration. All MEP responses were recorded throughout the experiment using LabChart software (ADInstruments, Bella Vista, NSW, Australia).

### Surface Electromyography Recordings

Surface EMG signals were recorded from the right and left RF using bipolar Ag/AgCl surface electrodes. The areas for electrode placement were shaved, scrubbed with an abrasive skin rasp to remove dead skin, and then cleaned with 70% isopropyl alcohol. The electrodes were placed on the muscle belly of the RF, midway between the anterior superior iliac spine and the superior border of the patella, with an inter-electrode distance of 2 cm (center to center). The reference electrode was placed on the right patella. All cables were fastened with tape to reduce movement artifact. The EMG signals, including MEPs and Mwaves, were amplified with a gain of 1000, band-pass filtered (13–1000 Hz), digitized at 2 kHz for 500 ms and recorded for offline analysis using PowerLab 4/35 (ADInstruments, Bella Vista, NSW, Australia).

### Data Analysis

The rmsEMG present in the right RF for the period 50 ms prior to stimulus delivery was recorded in mV and calculated automatically using the LabChart software. The mean of the five pre-stimulus values was then determined separately for each participant and each contraction intensity during both experiments.

The peak-to-peak MEP amplitude from each stimuli was normalized to individual participants’ *M*_max_ recorded immediately following each contraction intensity. The mean of the five responses recorded at each stimulation intensity (120% and 150%) was then calculated separately for each participant at each contraction intensity of the left quadriceps, to quantify corticomotor excitability.

The SICI_ratio_ was used to determine intracortical inhibition. This was calculated by dividing the mean paired-pulse MEP amplitude by the mean single-pulse (120%) MEP amplitude for each participant, and was performed separately for each contraction intensity of the left quadriceps. The SICI_ratio_ has an inverse relationship with intracortical inhibition, thus values close to zero represent higher intracortical inhibition, while values closer to one represent lower levels of intracortical inhibition.

In *Experiment 2*, silent period duration was measured by manually placing a cursor on each response to record the time elapsed (in ms) from the delivery of the stimuli to the return of pre-stimulus background EMG. The mean silent period duration for each participant at each left quadriceps contraction intensity was then determined for both 120% AMT and 150% AMT.

### Statistical Analysis

For each experiment, one-way repeated measure analysis of variances (ANOVAs) were used to detect differences in primary outcome measures for the right RF (corticomotor excitability, SICI_ratio_, silent period, *M*_max_, and pre-stimulus rmsEMG) between the contraction intensities performed with the left leg (0, 25, 50, 75 and 100% MVC). *Post hoc* pairwise comparisons with Bonferroni correction were used where significant main effects were found. All data was screened for normality using Shapiro-Wilk tests, and log transformations were applied to achieve normality when required (*Experiment 1*; corticomotor excitability and SICI_ratio_). Huynh-Feldt corrected values were reported when Mauchly’s test of sphericity was violated. Where *p*-values were close to significance (i.e., ≥0.05 but < 0.08), effect sizes were reported. All data is presented as a mean ± SD. SPSS version 22 was used for all statistical analysis, and statistical significance was set at the 0.05 level.

## Results

Thirteen of sixteen participants completed both *Experiment 1* and *Experiment 2*. One participant did not complete the *Experiment 1* due to a high RMT, and two participants did not complete the 100% MVC task in either experiment due to inability to reach required force output. Only participants with full data sets were included in the results.

### Experiment 1

#### Maximal Compound Waves

Mean values for *M*_max_ in the left RF and right RF following trials at each contraction intensity are displayed in Table 1. No significant difference in *M*_max_ between contraction intensity was detected (*F*_(4,52)_ = 0.808, *P* = 0.522).

**Table 1:** Maximal compound waves from the right and left rectus femoris (RF), and pre-stimulus rmsEMG values recorded from the right RF, for both experiments (Mean ± SD).

Experiment 1	0%	25%	50%	75%	100%
Left *M*_max_ (mV)	5.67 ± 1.66	6.01 ± 1.52	5.91 ± 1.77	6.27 ± 1.81	6.11 ± 1.74
Right *M*_max_ (mV)	5.64 ± 2.01	5.43 ± 1.89	5.56 ± 1.76	5.51 ± 2.01	5.46 ± 1.81
rmsEMG (mV)	0.003 ± 0.003	0.002 ± 0.001	0.003 ± 0.001	0.004 ± 0.002	0.009* ± 0.006
**Experiment 2**					
Left *M*_max_ (mV)	5.89 ± 1.50	5.84 ± 1.62	5.93 ± 1.69	6.06 ± 1.70	5.98 ± 1.63
Right *M*_max_ (mV)	5.83 ± 2.03	5.26 ± 1.60	5.25 ± 1.69	5.27 ± 1.56	5.33 ± 1.43
rmsEMG (mV)	0.024 ± 0.012	0.022 ± 0.010	0.028 ± 0.014	0.036 ± 0.019	0.061* ± 0.048

*Denotes significant difference (*p* < 0.05) from 0% maximum voluntary contraction (MVC).

#### Pre-Stimulus rmsEMG

Mean values for pre-stimulus rmsEMG recorded from the right RF are displayed in Table 1. Pre-stimulus rmsEMG of the right quadriceps remained under 0.004 mV during all left quadriceps contraction intensities except 100% MVC. A significant main effect for pre-stimulus rmsEMG between contraction intensities was detected (*F*_(4,176)_ = 4.289, *P* = 0.013). *Post hoc* pairwise comparisons with Bonferroni correction revealed a significant increase in pre-stimulus rmsEMG during 100% MVC (*p* = 0.041) when compared to 0% MVC. No other significant differences in rmsEMG were present (all *p* > 0.05).

#### Corticomotor Excitability

**Figure 2:**
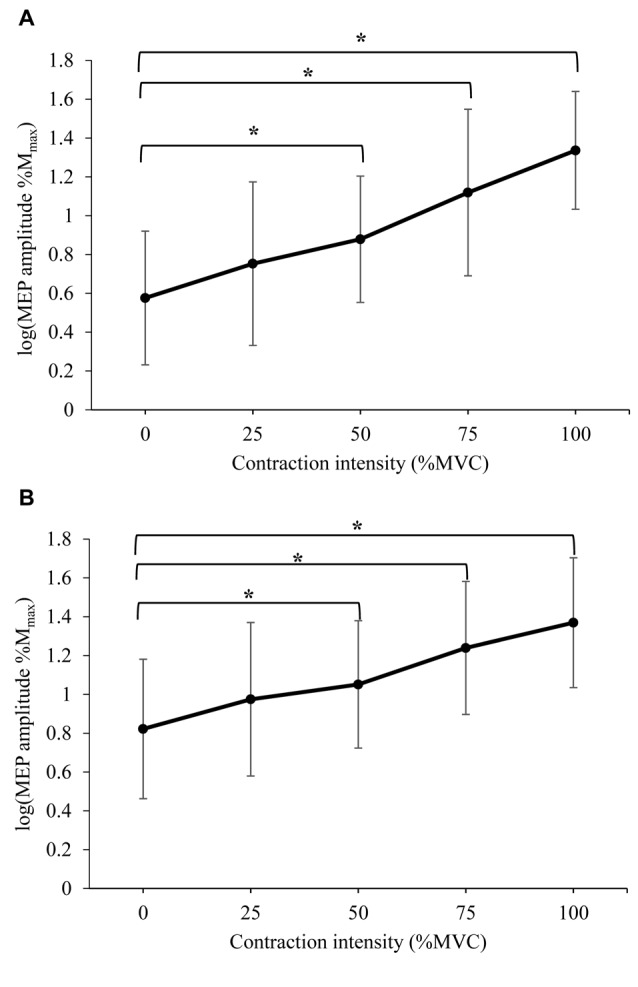
Mean ± standard deviation (SD) peak-to-peak amplitude of motor evoked potentials (MEPs) delivered at **(A)** 120% resting motor threshold (RMT) and **(B)** 150% RMT, recorded from the resting right RF during isometric contraction of the left quadriceps. *Denotes significant difference in *post hoc* pairwise comparison (*p* < 0.05). For MEPs collected at 120% RMT, contractions of 50%, 75% and 100% MVC produced a 96%, 300% and 425% increase in MEP amplitude, respectively. For MEPs collected at 150% RMT, contractions of 50%, 75% and 100% MVC produced a 66%, 155% and 229% increase in MEP amplitude, respectively.

The mean peak-to-peak amplitude of MEPs recorded following stimuli at: (a) 120% RMT; and (b) 150% RMT are displayed in Figure 2 (log transformed to achieve normality), and Table 2 (%*M*_max_). A significant difference in MEP amplitude at 120% RMT between contraction intensities was detected (*F*_(4,52)_ = 37.104, *P* < 0.000). *Post hoc* pairwise comparisons with Bonferroni correction revealed a significant increase in MEP amplitude during 50% MVC (*p* = 0.008), 75% MVC (*p* = 0.001) and 100% MVC (*p* < 0.000) when compared to 0% MVC. A significant difference in MEP amplitude at 150% RMT between contraction intensities was also detected (*F*_(2.856,37.126)_ = 30.035, *P* < 0.000). *Post hoc* pairwise comparisons with Bonferroni correction revealed a significant increase in MEP amplitude during 50% MVC (*p* = 0.035), 75% MVC (*p* = 0.004) and 100% MVC (*p* < 0.000) when compared to 0% MVC.

**Table 2:** Motor evoked potential (MEP) amplitudes (%*M*_max_) and short-interval intracortical inhibition (SICI)_ratio_ obtained during *Experiment 1* (Mean ± SD).

	0% MVC	25% MVC	50% MVC	75% MVC	100% MVC
120% RMT	5.01 ± 3.92	9.01 ± 9.77	9.84 ± 7.54	20.04 ± 18.09	26.32 ± 15.56
150% RMT	8.9 ± 6.76	13.52 ± 12.01	14.80 ± 11.99	22.69 ± 16.81	29.27 ± 16.91
SICI_ratio_	0.23 ± 0.21	0.42 ± 0.35	0.34 ± 0.22	0.40 ± 0.28	0.38 ± 0.17

#### Intracortical Inhibition

**Figure 3:**
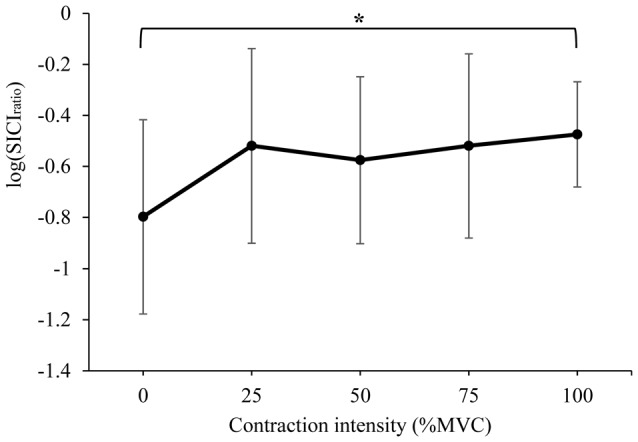
Mean ± SD short-interval intracortical inhibition (SICI)_ratio_ recorded from the resting right RF during isometric contraction of the left quadriceps. *Denotes significant difference in *post hoc* pairwise comparison (*p* < 0.05). Contractions at 100% MVC resulted in a 65% increase in the SICI ratio (from 0.23 to 0.38 of unconditioned MEP).

The mean SICI_ratio_ recorded from the right RF is displayed in Figure 3 (log transformed to achieve normality), and Table 2 (ratio data). A significant difference in SICI_ratio_ between contraction intensities was detected (*F*_(4,52)_ = 3.737, *P* = 0.009). *Post hoc* pairwise comparison with Bonferroni correction revealed a significant increase in SICI_ratio_ during 100% MVC (*p* = 0.022) when compared to 0% MVC.

### Experiment 2

#### Maximal Compound Waves

Mean values for *M*_max_ in the left RF and right RF following trials at each contraction intensity are displayed in Table 1. No significant difference in *M*_max_ between contraction intensities was detected (*F*_(4,52)_ = 0.605, *P* = 0.483).

#### Pre-Stimulus rmsEMG

Mean values for pre-stimulus rmsEMG recorded from the right quadriceps are displayed in Table 1. One-way repeated measures ANOVA detected a significant difference in pre-stimulus rmsEMG between contraction intensities (*F*_(4,176)_ = 5.227, *P* = 0.009). *Post hoc* pairwise comparisons with Bonferroni correction revealed a significant increase in pre-stimulus rmsEMG during 100% MVC (*p* = 0.039) when compared to 0% MVC. No other significant differences in rmsEMG were present (all *p* > 0.05).

#### Corticomotor Excitability

**Figure 4:**
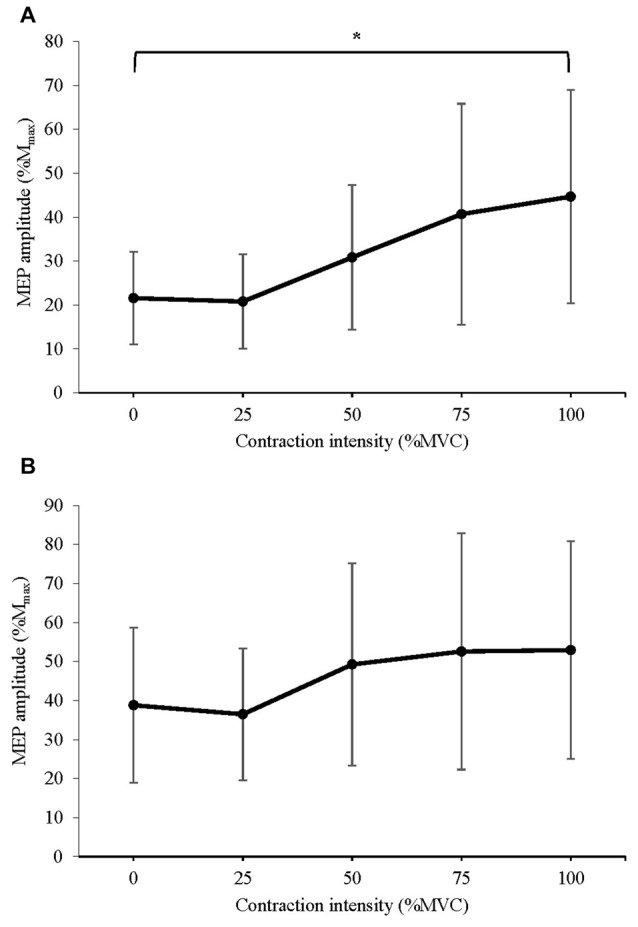
Mean ± SD peak-to-peak amplitude of MEPs delivered at **(A)** 120% active motor threshold (AMT) and **(B)** 150% AMT, recorded from the active right RF during isometric contraction of the left quadriceps. *Denotes significant difference in *post hoc* pairwise comparison (*p* < 0.05). For MEPs collected at 120% AMT, contraction at 100% MVC produced a 107% increase in MEP amplitude.

The mean peak-to-peak amplitude of MEPs recorded following stimuli at: (a) 120% AMT; and (b) 150% AMT are displayed in Figure 4. A significant difference in MEP amplitude at 120% AMT between contraction intensities was detected (*F*_(1.847,25.863)_ = 9.384, *P* = 0.001). *Post hoc* pairwise comparisons with Bonferroni correction revealed a significant increase in MEP amplitude during 100% MVC (*p* = 0.035) when compared to 0% MVC. No significant difference in MEP amplitude at 150% AMT was detected (*F*_(2.208,30.912)_ = 3.342, *P* = 0.053). The effect size was small (*η*^2^ = 0.193), and *post hoc* pairwise comparisons with Bonferroni correction revealed no trend for a significant difference between 0% and any other contraction level (all *p* > 0.10).

#### Intracortical Inhibition

**Figure 5:**
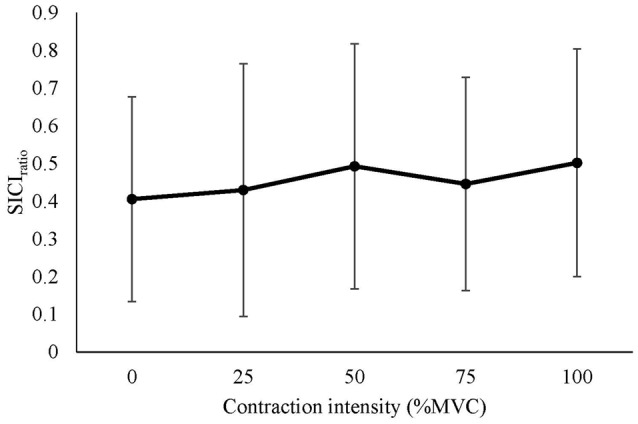
Mean ± SD SICI_ratio_ recorded from the active right RF during isometric contraction of the left quadriceps.

The mean SICI_ratio_ recorded from the right RF is displayed in Figure 5. There was no significant difference in SICI_ratio_ between contraction intensities (*F*_(4,56)_ = 0.471, *P* = 0.756).

#### Silent Period Duration

**Figure 6:**
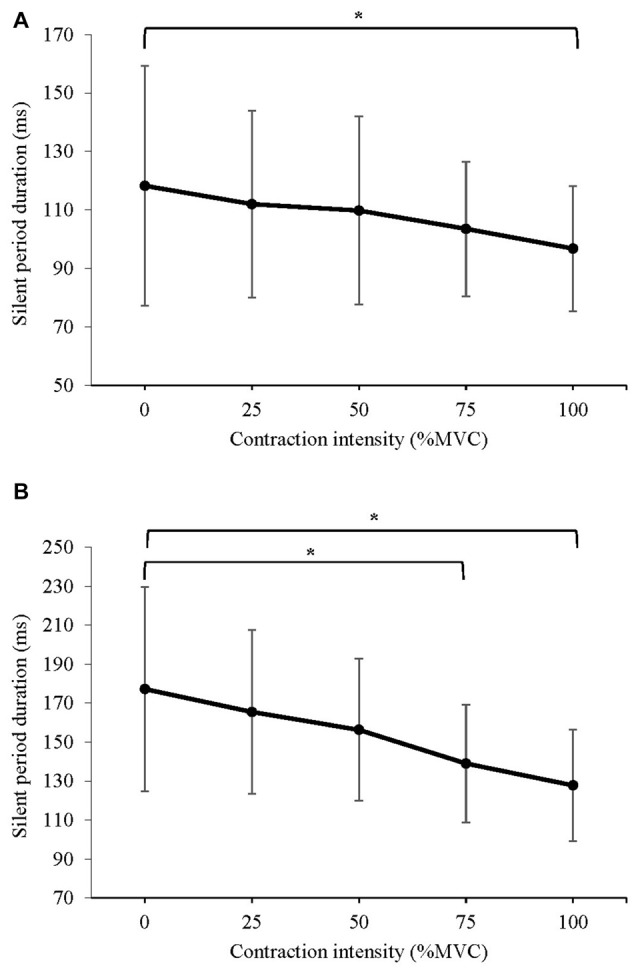
Mean ± SD silent period duration of MEPs evoked at **(A)** 120% AMT, and **(B)** 150% of AMT. For MEPs collected at 120% AMT, there was an 18% reduction in the silent period during 100% MVC. For MEPs collected at 150% AMT, contractions of 75% and 100% MVC produced a 22% and 29% reduction in the silent period, respectively.

The mean silent period duration of MEPs evoked at: (a) 120% AMT; and (b) 150% AMT are displayed in Figure 6. A significant difference in silent period duration at 120% AMT was detected (*F*_(1.660,21.584)_ = 4.275, *P* = 0.033). *Post hoc* pairwise comparison with Bonferroni correction revealed a significant decrease in silent period duration during 100% MVC (*p* = 0.028) when compared to 0% MVC. A significant difference in silent period duration at 150% AMT was detected (*F*_(1.480,19.240)_ = 6.067, *P* = 0.014). *Post hoc* pairwise comparison with Bonferroni correction revealed a significant decrease in silent period duration during 75% MVC (*p* = 0.021) and 100% MVC (*p* = 0.016) when compared to 0% MVC.

## Discussion

Our results demonstrate that cross-activation of the iM1 during unilateral contractions of the lower limb occurs at isometric contraction intensities of 50% MVC and above. We observed a linear, monotonic increase in the magnitude of cross-activation as contraction intensity increased, despite randomization of testing order. The effect of isometric contractions on iM1 inhibitory circuits was less pronounced, with significant reductions in SICI only observed during maximal isometric contractions (100% MVC) when the inactive limb was at rest. The reduction in the silent period duration of the right leg observed during 75% and 100% MVC of the left leg suggests that the reduced inhibition along the corticomotor pathway of the inactive limb may also contribute to the cross-activation effect. Unchanged *M*_max_ responses throughout the experiment in both the resting and active RF suggest peripheral muscle fatigue did not contribute to the findings. Importantly, maximal contractions of the left quadriceps caused an increase in pre-stimulus EMG of the right resting RF. This co-contraction occurred even when participants were reminded to relax the contralateral limb, and during trials with full relaxation (*Experiment 1*) as well as trials with low level background activity (*Experiment 2*). This may indicate that the spill-over of iM1 activity during maximal efforts is sufficient to activate the entire corticomotor pathway, reaching the peripheral muscle at levels detectable by rmsEMG, although not resulting in visible movement.

### Cross-Activation

A linear, monotonic increase in the amplitude of MEPs obtained from the resting right RF was observed as contraction intensity of the left quadriceps increased. This increase in excitability of the corticomotor pathway reached statistical significance at contraction intensities of 50% MVC and above. This relationship between contraction intensity and cross-activation is a novel finding in the lower limb, where the effect of low-intensity contractions has not been previously investigated. In agreement with our findings, one previous study has reported increased excitability of the resting RF and TA during contractions at 75% of maximal EMG (Chiou et al., 2013a). However, investigation of cross-activation in the upper limb have produced conflicting results, reporting significant increases in MEP amplitude during lower intensity contractions, such as 30% MVC (Perez and Cohen, 2008) and 10% MVC (Stedman et al., 1998). While our study did not directly compare the magnitude of cross-activation in the upper vs. the lower limbs, when taken together with previous findings, our results indicate that lower limb musculature may require greater contraction intensities in order to induce cross-activation. This variance may be due to the difference in functional requirements of upper and lower limb musculature (Chiou et al., 2013a). For example, the gross nature of lower limb movements, as well as their involvement in reciprocal movements such as gait, may require a lesser degree of bilateral facilitation of the homologous muscle than coordinated movements in the upper limb. In addition, early TMS research suggested that the higher threshold stimulation required to evoke responses in the lower limb indicated a lower density of corticomotor projections when compared to muscles of the upper limb (Brouwer and Ashby, 1990). This was especially true for proximal lower limb muscles such as the RF (Brouwer and Ashby, 1992). Despite this, we observed MEP facilitation of up to 425% during 100% MVC contractions, indicating that unilateral contraction of the lower limb does indeed produce a profound effect on the excitability of the contralateral homologous muscle.

### SICI and Silent Period Duration

We observed a 65% mean reduction in the SICI_ratio_ of the iM1 during isometric unilateral contractions at maximal (100%) intensity, however, submaximal contractions did not produce a significant release of SICI. It should be noted that maximal contractions caused co-contraction of the resting limb, detected via an increase in rmsEMG prior to stimulus delivery, which may have contributed to the reduction in SICI. These findings are in contrast to the majority of evidence obtained from upper-limb studies, where the release of SICI in the iM1 during unilateral contractions appears to be more pronounced (Muellbacher et al., 2000; Perez and Cohen, 2008). For example, SICI was nearly abolished completely during isometric contraction of the FCR at 70% MVC (Perez and Cohen, 2008), and during maximal contraction of the abductor pollucis brevis (Muellbacher et al., 2000). Our data showed that even during maximal contractions of the contralateral quadriceps, large suppression of the conditioned MEP remained (38% of unconditioned MEP). Chiou et al. (2013a) also reported a reduction of SICI in the RF during contractions of 75% EMGmax when compared to rest, with a similar degree suppression of the conditioned MEP remaining (43% of unconditioned MEP). In the same study, a greater reduction in RF SICI was reported when contralateral contractions of the FCR were performed (63% of unconditioned MEP), with the authors discussing the role of functional inter-limb co-ordination between the upper and lower body as a possible reason for this finding (Chiou et al., 2013a). Certainly, our results suggest that submaximal unilateral contraction has little effect on SICI of the homologous muscle representation, which may be explained by the need for reciprocal inhibition of the opposite limb during the out of phase movements that are typically performed by the lower limbs (i.e., gait).

Another possible reason for observing a comparatively small effect on SICI of the iM1 in *Experiment 1* may be due to the methodological approach, whereby participants were told to avoid or suppress movement in the resting leg. This is likely to cause volitional inhibition, which has previously been shown to increase cM1 SICI during tasks where stop signals were provided immediately before anticipated movement (Coxon et al., 2006). If volitional inhibition was the reason for high levels of intracortical inhibition in the resting leg during contralateral submaximal contractions, it would be expected that this effect would be abolished during *Experiment 2*, where light background activity was performed. This was not the case, with the mean conditioned MEPs ranging from 40% to 50% of the unconditioned MEP amplitude, and no significant difference in SICI ratio during any of the unilateral contraction tasks. Overall, our results suggest that unilateral contractions in the lower limb have a less pronounced effect on iM1 SICI of the homologous muscle than unilateral contractions of the upper limb.

Interestingly, we observed a linear reduction in silent period duration as contraction intensity in the contralateral limb increased, reaching significance at 75% MVC for MEPs evoked at 150% AMT. A previous study has reported lower levels of variability and greater homoscedasticity in the silent period duration of MEPs evoked at intensities >130% AMT (Damron et al., 2008), which was supported by our data (see Figure 6). A reduction in silent period duration represents a net decrease in corticomotor inhibition, however, the segmental level at which inhibition decreases cannot be determined from examining silent period alone. When considered alongside the unchanged measure of SICI, these results indicate that unilateral contractions produced a reduction in inhibition downstream of the iM1, for example, reduced gamma-aminobutyric acid B (GABA_B_) activity at the level of the spinal cord.

### Co-Contraction during Maximal Efforts

Despite conscious efforts to maintain relaxation (*Experiment 1*) and to keep low level contraction during leg extension constant (*Experiment 2*), participants were unable to produce statistically similar pre-stimulus rmsEMG in the right RF during maximal (100% MVC) contractions of the left leg (see Table 1). The majority of previous studies investigating cross-activation with TMS have used 0.025 mV as the cut off point for background EMG in the resting limb, discarding trials where background activity exceeded this value (Muellbacher et al., 2000; Hortobágyi et al., 2003; Perez and Cohen, 2008; Chiou et al., 2013a,b). We employed a similar strategy in *Experiment 1*, discarding any trials that exceeded 0.025 mV and asking the participant to repeat the trial. Further to this, we ran a repeated measures ANOVA on the rmsEMG recorded in the 50 ms epoch prior to stimulus delivery, in order to detect any minor changes in background muscle activity that may potentially affect MEP responses. In *Experiment 1*, we found that despite meeting the criteria of <0.025 mV as a raw EMG signal, there was, in fact, a significant statistical difference in right leg pre-stimulus rmsEMG during left leg 100% MVC (see Table 1). Similarly, a significant increase in pre-stimulus rmsEMG was observed in *Experiment 2* during maximal contractions. No observable mirror movements were reported in either experiment.

The increase in background EMG of the resting limb during demanding single limb tasks has been frequently observed in the upper limb, and has been termed “motor irradiation” (Cernacek, 1961; Hendy et al., 2012). We asked participants to minimize these effects in order to remain consistent with previous literature, and also to provide a more simple interpretation of the neurophysiological response to single limb contractions. Instead, we showed that motor irradiation in the lower limb was unavoidable during maximal isometric contractions, and hence we propose that this very low level involuntary activity represents further evidence for an increase in excitability of the “resting” corticomotor pathway, essentially producing the same (and in this case, desirable) outcome. Further examination of this phenomenon in the lower limb is warranted.

### Significance

The findings from this study are of importance when developing long-term training protocols to induce cross-education of strength. The use of unilateral training to supplement rehabilitation following single limb immobilization (Pearce et al., 2013) and injury (Magnus et al., 2013) has been implemented in the upper limb, but to our knowledge, such studies are yet to be conducted in a lower-limb paradigm. Our results indicate that unilateral training should be implemented at the highest intensity tolerated by the participant, in order to maximize activation of the iM1 and excitability of the corticomotor pathway innervating the unexercised muscle. Small increases in background muscle activity appear to be unavoidable during maximal contractions of the quadriceps, however, the presence of this motor irradiation is likely to have very minor, but potentially positive effect on the overall magnitude of cross-education. In a unilateral training setting, it seems counterintuitive to reduce motor irradiation by instructing the patient to “relax” the unexercised muscle, potentially increasing volitional inhibition and reducing the net magnitude of cross-activation. Current experimental protocols designed to minimize motor irradiation in the resting limb may need to be modified when utilizing cross-education training in injury rehabilitation settings, as the nature and magnitude of motor irradiation in the presence of injury or pain is not currently known, and may differ from observations in healthy participants.

### Strengths and Limitations

Muscle fatigue has been shown to increase the magnitude of cross-activation, and increase levels of background EMG in the resting muscle (Arányi and Rösler, 2002). Given the number of isometric contractions required to complete our protocol, we anticipated that fatigue may potentially confound our findings. In order to minimize this, the order of contraction intensity was randomized, and M-waves were assessed after each task. Importantly, our results showed no change in *M*_max_ of either the right or left RF throughout the experiments, indicating that the protocol did not produce peripheral muscle fatigue. With the intention to minimize the likelihood of fatigue, we used a relatively low number of TMS pulses to determine corticomotor plasticity (five pulses per condition, where previous studies have used up to 30; Chang et al., 2016), delivered at low (120% AMT) and high (150% AMT) intensity, rather than MEP recruitment curves. While this presents as a potential limitation, the use of five pulses was strategically selected to provide adequate MEP data while minimizing fatigue (Groppa et al., 2012).

Since transcallosal pathways between the hemispheres contribute to the net corticomotor output of the M1 (Avanzino et al., 2007; Lee et al., 2007), it is likely that the IHI would be altered during unilateral contractions. Indeed, studies investigating IHI during unilateral contractions of the upper limb have demonstrated decrease in IHI during isometric contraction intensities of 30% and 70% (Perez and Cohen, 2008) and both concentric and eccentric contractions of 90% MVC (Howatson et al., 2011). We were unable to measure this in the lower limb musculature, due to the close proximity at which the left and right RF motor representations are located anatomically, preventing the concurrent placement of two separate coils over the right and left RF optimal site. We acknowledge this as a notable limitation in this study, and in the investigation of IHI in lower limb muscles in general. In addition, we acknowledge that the analysis of silent period duration using a manually placed cursor must be noted as a limitation of this study.

Finally, given our results indicate that reduced inhibition downstream of the iM1 may play a larger role in the increased net corticomotor excitability of the right RF, investigation at segmental levels is required to further elucidate the underpinning mechanisms. Future studies should measure H-reflexes and MEPs elicited at the cervicomedullary junction during unilateral contractions of the lower limb. While such investigation has been conducted in the upper limb (Hortobágyi et al., 2003), our results suggest that mechanisms for cross-activation in the lower limb may differ.

## Conclusion

We observed a linear increase in corticomotor excitability of resting RF during increasing isometric contractions of the contralateral limb. Unilateral contraction intensities of 50% MVC and greater result in significant increases in corticomotor output to the resting homologous muscle. These findings suggest that exercise protocols designed to induce cross-education strength gains in the lower limb should be applied at an intensity of at least 50% MVC, with higher intensities preferable in order to maximize stimulus of the corticomotor pathway that activates the resting or unexercised limb. Furthermore, we conclude that a reduction in SICI occurred only during maximal isometric efforts, and may not play a major role in the net output of the iM1 during unilateral contractions. Instead, reductions in the silent period duration during 75% and 100% MVC efforts suggest subcortical and spinal inhibition may contribute to cross-activation of the inactive limb.

## Author Contributions

AMH and W-PT developed the conceptual basis and experimental design for this study. Data collection and analysis was conducted by AMH and LC. Interpretation of results and manuscript preparation was carried out by AMH.

## Conflict of Interest Statement

The authors declare that the research was conducted in the absence of any commercial or financial relationships that could be construed as a potential conflict of interest.
